# Atlanta Academy of Medicine

**Published:** 1876-07

**Authors:** R. W. Westmoreland

**Affiliations:** Reporter


### Atlanta Academy of Medicine.

R. W. WESTMORELAND, M.D., REPORTER.

ATLANTA, May 16th, 1876.

Meeting called to order by the Vice-President, Dr. J. G. Westmoreland.

Dr. Jno. M. Boring exhibited a stone taken from a man 76 years old. This case he had treated for two years, during which time he was called on several times to relieve him of retention of urine. The patient would never allow him to introduce a catheter until last Sunday, the urine having been retained then 48 hours. After many efforts he failed to introduce the instrument, and Dr. W. F. Westmoreland was called in consultation. An operation was decided on, the patient, though old, being otherwise stout and in good health. On Tuesday morning saw him, and discovered that he was growing rapidly weak; in the evening he was found to be so low that it was deemed useless to operate. Thursday evening he died, and on post-mortem the stone exhibited was removed, and found to weigh eleven hundred and sixty grains. Remarked that he would be glad to hear from Dr. Westmoreland on the case.

Dr. W. F. Westmoreland remarked that Dr. B.'s case was attended with several peculiarities. The old gentleman had paroxysms of more or less complete retention of urine, and died from suppression, resulting in uræmia. On his first visit he found him in one of his paroxysms of retention. The bladder was distended with urine to the extent that it could be felt as a hard, unyielding tumor, the size of a child's head, above the pubis. Notwithstanding the frequent and terrible efforts to empty it, no urine was discharged, nor had there been for twenty-four hours preceding. A small catheter was introduced and passed readily to the bladder, where it was arrested by coming in contact with the stone, or what he supposed at first was a number of stones. Considerable force was exercised, and the instrument manipulated by carrying the beak in various directions, without, however, being able to dislodge the stone, or to pass the instrument by it. The idea then suggested itself that probably one might be passed over a filiform bougie. This was attempted, but though it would pass through the prostate gland, as was determined by the finger in the rectum, it would not go farther. An English catheter, No. 12, was introduced to the stone, and tepid water to the amount of a gill was thrown in, the object being to distend the neck of the bladder that grasped the stone, or to displace the stone. Finding it impossible to pass the catheter by the stone or empty the bladder, a small silver catheter was passed in its place, which succeeded in passing the stone, and the accumulated urine withdrawn. The patient being stout and otherwise in good health, an operation was decided on for next day. The day being unfavorable, it was deferred another day. On visiting him the next day, it was found that he had passed no urine for twenty-four hours, nor had he made an effort to do so. Examination revealed the fact that no urine had been secreted. He had been in a comatose condition for ten or twelve hours, and could not be aroused. He died the same night, evidently of uræmia. The family consenting, a post mortem was had eight hours after death. An incision, as in superpubic operation for stone in the bladder, was made in the abdominal walls, and cause of difficulty in introducing catheter and retention of urine revealed. The stone, as you see of large size, was firmly imbedded in the neck of the bladder, not in the urethra but the neck, forming a pocket, which held it in position. An ounce or two of urine, with perhaps as much mucus, was found above the stone. So firmly was the stone grasped, even after death, that some difficulty was experienced in removing it from its pocket with a pair of forceps. It is certainly unusual to have retention of urine in stone, unless the calculi enter the urethra. Again, the sudden and unexpected termination of this case, is peculiar and unusual.

Dr. Westmoreland also reported a case of elongated cervix uteri in a patient 18 years old. She had complained of "falling of the womb," and it had the appearance of true procidentia, but examination showed the real condition. The uterus measured 5½ inches; the neck projected an inch or more through the labiæ. There was no displacement of body of the womb. The ecraseur was used to amputate. About an inch and a half of the neck was removed without the least hemorrhage. No anæsthetic was administered until the patient, from the shock, became pulseless, with symptoms resembling uterine colic when stimulating fluids are injected into the womb. These symptoms came on before the neck was severed. Ether to complete anæsthesia promptly relieved the shock, and the operation was completed without further accident. It is now eighteen days since the operation, and the woman is well. Always operates with ecraseur in such cases and has never had either primary or secondary hemorrhage to occur.

Dr. Taliaferro spoke of the frequency of elongation. Thinks it is often taken for procidentia. He has seen several instances where such was the case. He has never used the ecraseur. He operates with knife and usually has secondary hemorrhage. So impressed has he become of its probability that he makes it a special point to guard against it.

Dr. W. F. Westmoreland remarked that he has never, out of a number of such cases, had either primary or secondary hemorrhage result from the use of the ecraseur. He thinks that, should he ever have occasion to abandon this instrument, that he would prefer to ligate the arteries, to following the books, which say tampon with some styptic application

Dr. Connally mentioned a case in corroboration of Dr. Taliaferro's remarks, where it was supposed to be procidentia, but he found it to be elongation of cervix. The patient was thirty years old, been married ten years, and had no children. Has been troubled this way five years, but never confined to her bed. Menstruates regularly. The cervix protruded two inches, the whole measurement of the uterus being 5½ inches. The cervix was smaller where it protruded from labiæ, as if it were pressed between them. Assisted by Dr. Taliaferro, one inch was removed by the flap operation; flaps secured by silver sutures. Considerable difficulty was experienced in bringing the flaps together on account of the indurated condition of the tissue. Some primary hemorrhage, though not a great deal. Considerable secondary hemorrhage.

Dr. John M. Johnson mentioned a similar case which he cured with mur. tinct. iron and mur. of ammonia. Used tinct.

of iron for accompanying leucorrhœa. The condition disappeared by gradual retraction. Fifteen grains of the muriate ammonia, three times a day, was used. Treated the case five weeks.

Dr. Connally, in reference to Dr. W. F. Westmoreland's suggestion to ligate, remarked that in his case it would have been difficult to do on account of the induration.

Dr. John M. Johnson spoke of the cure of goitre by the external and internal use of mur. ammonia. Supposed that other glandular and fibrinous affections subject to the same influence. Has seen cases of fibroid tumors of the uterus benefited and, as he believed, even cured by this remedy.

Dr. Todd agreed with Dr. Johnson in reference to the constringing effect of tincture of iron in certain conditions of the uterus. Inquired if any member had used mur. ammonia in neuralgia. Believed it very good for such affections. Inquired of Dr. Calhoun as to its use in Germany.

Dr. Calhoun remarked that it was a very favorite remedy in Germany; did not know for what affections it was specially used.

Dr. John M. Johnson remarked that he had derived great benefit from its use in conjunction with blisters and tonics in a case of sciatica. Cured the case in two months. It returned in eight years. It was then treated by electricity. Has used it in neuralgias, with tonics, with benefit.

Dr. Taliaferro remarked that as in elongation of cervix uteri we have hyperplasia of connective tissue, which, to be corrected, must be by fatty degeneration and absorption; can't understand the rationale of administering mur. ammonia. As for its use in fibroids, &c., he could not see how it would benefit them.

Dr. John M. Johnson replied that he had not succeeded in curing all the fibroid tumors by the remedy. Suggested that Dr. Taliaferro try it if he would have his skepticism removed.

Dr. W. F. Westmoreland inquired of Dr. Johnson what he meant by goitre getting well; if it entirely disappeared? He asked for the reason that he had tried all remedies that he had heard spoken of. With some he would cause a decrease of the tumor, though he has never succeeded in causing its entire disappearance.

Dr. John M. Johnson replied that as far as he knew they entirely disappeared.

Dr. J. T. Johnson remarked that he noticed a case in the College Clinic that had been treated for a number of months; he could never discover any diminution.

Dr. Todd asked Dr. Taliaferro if he had used ergot for fibroid tumors in the uterus.

Dr. Taliaferro replied that he had, but the cases had not been long enough under his observation to decide upon its efficacy. Has been favorably impressed, however.

Dr. John M. Johnson, in advocating ergotine for impotency, mentioned a case he had benefited by its use.

At the conclusion of his remarks on the history of the case and its treatment, the Academy adjourned.

---

ATLANTA, GA.. May 23, 1876.

Meeting called to order, President, Dr. Miller, in the chair.

Dr. Ford, Essayist for the month, read the following paper:

"Artificial Dilatation of the Os Uteri as a means of hastening natural labor."

I have chosen this subject, not because I was conversant with it, for in all of my teachings not one word have I heard mentioned of artificial dilatation of the os uteri as a means of hastening natural labor. Nor do I intend to try to decide this question, for my age and experience would not allow it. But shall endeavor, as far as possible, to give the views of all the authors within my reach who have written on this subject; and I shall not slight those who oppose it, nor neglect those who are absolutely silent as to the means now under consideration. But it is my desire, as far as in my power lies, to bring the evidence, pro and con, to bear upon this subject, and leave the Academy to decide it.

If it deserves the attention ascribed to it by the older authors as a useful expedient and as a means of cutting short the pangs of human suffering, which would last hours otherwise, it certainly deserves some attention at the present time. After I shall have finished I will be glad to hear the opinion of every member of the Academy as to whether they are accustomed to let nature take its course or attempt to interfere.

In the *London Lancet*, of 1852, I find an article written by James Gilmore, in which he says: "Artificial dilatation of the os uteri does uot seem to have engaged the attention of British obstetricians so much as it deserves, as a means of accelerating natural labor, and shortening the pangs of a very painful process in the animal economy. I have often been surprised at the silence of modern midwifery writers on this subject, as it could scarcely have escaped their notice in an extensive practice; and on referring for information to the older authors we occasionally find mention made of it, though it is not dwelt upon with that attention which the subject demands."

If my memory is correct, I only recollect one article written on this subject in the pages of the *Lancet* for the last seven years, by Dr. Green, of Dublin, and then it does not seem to have elicited any remarks from the profession.

This is the more strange as medical practitioners are keenly alive to any method alleviating human suffering, provided the proposition bears the stamp of common sense, and is founded on these data. Now, why has artificial dilatation not obtained sufficient notice by the profession? Is it because any interference with natural labor is considered unnecessary? Is it owing to some modern writers not approving of the method under discussion? Is it owing to other authors being silent on the subject? I cannot tell.

Now, in Blundell's elaborate work little notice is taken of artificial dilatation in lingering labor; on the other hand, every manual interference is condemned; and throughout the entire book one maxim is ever kept in view, viz: "Meddlesome midwifery is bad," this he calls the first article of the obstetric creed.

"No practitioner would hesitate in cases of protracted labor arising from rigidity of the os uteri or vaginal passage, to use the remedies at our disposal for benefiting such a condition. In my own practice, he says, "which is not small, I seldom use the ergot, but invariably, when requisite, practice artificial dilatation; and during the last five years I have not had to treat a single symptom arising from such a proceeding. I have not had a case of inflamation of either cervix uteri or vagina, though many writers, who are opposed to artificial dilatation, seem to think that if we attempt any means of expediting delivery we bring on all manner of inflammatory and other dangerous maladies."

We find that even Celsus was not altogether ignorant of artificial dilatation, for we find him stating that "in cases where a dead fœtus is contained in the womb, the physician is to insert his hand, finger by finger, into the womb to expedite the delivery."

Daventer, speaking of the conditions demanding artificial dilatations, uses these words:

"Sometimes it happens that the pains which seemed to increase sufficiently in the beginning are afterwards much diminished, or altogether cease, which most commonly happens for want of sufficient help." He then goes on to remark "that an expert practitioner in midwifery may do much good by *stirring up* the pains with an experienced hand, and in other parts of his work he imagines that his method of artificial dilatation will supersede the use of instruments."

Chapman's words are: "Sometimes there shall be all the usual symptoms of labor, the pains strong and true, and the head of the child very low, bearing forcibly down every pain, yet the os tincæ shall be very little dilated, and that after many hours of strong pain. Here nothing more is to be done than with the fingers to dilate and thrust back the ring or circle, if I may so speak, which the os tincæ (now very thin) makes upon the head of the child."

He gives us very plain directions as regards the mangement of lingering cases. He remarks: "It will sometimes be found necessary to insinuate the fingers, with the flat of the hand between the head and os internum, for when this precaution is not taken in time the os internum is frequently pushed before the head, even through the os externum. And again, he relates a case of rigidity of the os uteri, where bleeding and other remedies were used, and the delivery accomplished by pushing up the fingers round the head, which at last glided easily along and was delivered."

Denman entertains different views, opposing any interferference at all. His own words will best explain his method of argument:

"Whether a short or long time be required for this purpose (dilatation of the os uteri), it is the duty of the practitioner to abstain from interfering in this part of the process. It may sometimes be necessary to pretend to assist, with the intention of giving confidence to the patient, or composing her mind. But all artificial interposition contributes to retard the event so impatiently expected, by changing the nature of the invitation and the action thereon depending."

Burs seems to lay aside all timidity which had hitherto occupied the mind of the older authors, and boldly pronounces upon the very great importance and benefit arising from artificial dilatation. His words are: "Forcible and irritating dilatation of the os uteri, when it is not productive of dangerous consequences, is apt to occasion irregular or spasmodic action of the uterus. Two circumstances are necessary to render it safe, the os uteri must be lax and dilatable, and the dilatation must be gradually and gently effected during the continuance of a natural pain. If attempted in the absence of a pain, and especially if attempted so as to give pain, it is apt to excite particular spasmodic action, and, under any circumstance, violent or forcible dilatation, besides injuring the uterine action, may lay the foundation of future disease. It is best done by pressing on the anterior edge of the os uterus during a pain, with two fingers, with such moderate force as shall not give additional pain and shall appear to excite the natural dilatation as much as to produce mechanical opening. By doing this for several pains in succession, or occasionally during a pain, at intervals, according to the effect produced and the disposition to yield, we shall have the os uteri completely dilated. This same author says it is a good general rule to effect the dilatation of the os uteri within ten or twelve hours at the farthest, from the commencement of regular labor. Taking away, at a favorable time, the resistance afforded tends to excite efficiently the action of the uterus and promotes labor. If the os uteri be lax, and especially if its edges be soft and thin and the orifice considerably dilated, the same effect may be produced on it by this practice that would be in cases of greater rigidity by venesection, for both excite labor by diminishing resistance. The more the os uteri is dilated beyond the size of half a crown, the more beneficial, *cœterus paribus* will be the practice; on the other hand, when the os uteri is firm and little dilated, and the other soft parts are rigid, this practice, so far from being useful, is hurtful and dangerous."

Dr. Gilmore remarks: "It was a maxim of the late Dr. Hamilton, of Edinborough, that, unless under very peculiar circumstances, the first stage of labor should be completed in twelve hours from the commencement, and if not so, that measure should be forthwith adopted, to secure the object as speedily as possible, as the protraction was productive of many untoward circumstances very unfavorable to the patient.

"In this, the same writer says, I fully coincide. During the last five years he has conducted more than 1,700 labors, and has invariably tried to follow this rule—to finish the first stage in twelve or fourteen hours. Be it remembered that I now only allude to tedious labor, and, provided, as Burs remarks, the pains must be continuing so often and so decidedly, that the patient can be said to be in actual labor all the time."

Gooch is in favor of artificial dilatation. He says: "I then applied two fingers against the edge of the os uteri next the symphysis pubis, and pushed it up at the time of pain, and kept it up after the pain was gone off. At the next pain I pressed the os uteri still higher, and repeated the same proceeding at about a dozen pains, when the os uteri slipped quite over the head of the child, and the labor was soon over."

I have consulted a class of obstetrical writers who are absolutely silent as to the subject now under consideration. Among the number may be enumerated Velpeau, Rigby, Churchill, Tucker, Ramsbotham, Chally, Dewees. The works now most in vogue are Meigs's and Leishman's, and in these there is nothing said about artificial dilatation of the os uteri as a means of hastening natural labor; per contra, every interference is condemned that tends to assist natural labor.

Although Leishman is opposed to any interference in natural labor, he admits the power of frequent examinations to excite uterine action.

Under the head of Uterine Inertia, we find him using the following words:

"The reflex activity of the uterus is often aroused by digital examinations which seem to excite the nerves of the cervix, or those which are distributed in some abundance to the tissues of the perineum. Free examination of those parts, therefore, which under ordinary circumstances is to be condemned, may be practiced without hesitation should the uterus show any symptom of repose."

James Gilmore, of Glasgow, has laid down indications by which all practitioners may be governed:

"The first class of cases in which artificial dilatation may be beneficially tried are those in which the waters have been some time discharged, the os uteri slightly open, lax, soft, and thin, with the head resting closely on the os, the patient having been several hours in this state, with the pains either unimportant, not exerting sufficient vis a tergo force on the os uteri to dilate it, or being inefficient though coming on every five minutes. In such cases as these, especially if it be a secondary or succeeding birth, artificial dilatation is both safe and advantageous, and, if done with due caution, will give no additional uneasiness, but will cause the os to clear the head very speedily, and terminate the labor, probably, in one or two hours; whereas, without such assistance, a whole day would have been spent over its completion and the patient left in a much more precarious state as regards her recovery."

"A second class of cases are those where the os uteri is projecting, thick, and yielding, the pains lingering, the waters discharged or not, the patient being several hours in labor.

"A third class of cases are those where the waters have been sometime discharged, the os uteri considerably dilated, with its edge thin and dilatable, no heat of the vagina or other symptom of irritability, or inflammatory state of the system, with a portion of the scalp protruding through the opened os, in a wedge-like shape, and the pains slow and ineffective. In this condition artificial dilatation will be most beneficial, often terminating the first stage rapidly, and likewise rousing the dormant powers of the uterus so efficiently as to finish the labor entirely in a very short time. The dilatation in these cases is effected as follows: The index finger is rested on that portion of the os uteri, adjacent to the symphysis pubis, commonly named its anterior edge or lip and gentle pressure made on it, in an upward direction, during a pain, as if we wished to push the rim over the child's head; we shall soon find the pains becoming more frequent and effective, the head gently (though rapidly) passing through the os, and the labor soon over.

The writer of this paper, as before stated, does not pretend to decide this mooted question, but submits it to the Academy for consideration.

Upon conclusion of the Essay of Dr. O. R. Ford, Dr. Taliaferro remarked that he was not in favor of any interference with natural labor. In such cases the progress and manner of dilatation adopted by nature are all that should be desired.

Dr. Boring, like Dr. T., favored non-interference with the natural process, though he has at times resorted to artificial dilatation. Is satisfied he never derived any benefit from such attempts, and is, therefore, an advocate of leaving it to nature and time.

Dr. Rauschenburg, considering the process of dilatation as a mechanical one carried on by the insinuating contrivance of the membranes, thought, in the absence of this agent, that artificial dilatation would be very beneficial and opportune. Usually accomplishes this by introducing a finger, and by a circular motion dilates gradually, until all the fingers and even the hand may be introduced. Never dilates in rigidity, but bleeds instead.

Dr. Taliaferro remarked that the only stage of labor in which artificial dilatation could be practiced was in the *first stage*, and that certainly less interference was demanded in this stage than in any other. This was the stage of dilatation. Nature might effect this process slowly or rapidly. It may consume six hours or twelve, or twenty-six hours, or longer. At any rate, the time consumed in the first stage of labor is immaterial, so that it is not too rapid. In the process of a slow dilatation nature softens and lubricates the parts preparatory to the more critical stage of labor where dilatation has already been effected. The only interference with the first stage of a natural labor at all admissible, is the administration of an opiate to give the patient a period of rest from the harrassing pains of dilatation.

Dr. J. G. Westmoreland considered the direction in which the fœtus was forced, from some obliquity of the uterus, as a point requiring artificial interference. Is in the habit of resorting to ergot when it is necessary to hasten natural labor.

Very often in a weak action of the longitudinal fibers, as compared to the circular ones, dilatation is retarded. Ergot, by its action on these longitudinal fibers, excites them to promote the dilatation.

Dr. Nunn thought three things were to be taken into consideration in this question:

1. What is natural labor?
2. What would be to the safety of the child?
3. What to the mother?

The state of innervation arising from numerous social errors prevents very often what may strictly be called a natural labor. Civilization, in this respect, has proven a bane not easy to be gotten rid of. The displacements often resulting from this innervation he attempts to correct by dilatation of the os uteri. But at all times where he interferes to hasten labor, he gauges his efforts by the strength of child, as found out by auscultation and other means. If he finds the fœtus weakening he hurries the more. As for the use of ergot, his only use of it is to prevent post partem hemorrhage; is afraid to use it in the process of labor because you cannot control its action, and thereby endanger the life of the child. Coincides with Dr. Rauschenberg in substituting the finger for the membranes when from their rupture they do not serve their mechanical purposes in dilatation.

Dr. John M. Johnson thought manipulation of a rigid os uteri with the finger very beneficial in promoting dilatation. In displacement of the os where it sinks down so that the force of the contraction are not exerted in the proper axis, by raising it up he assists it in its expansion.

Dr. Taliaferro, referring to Dr. Nunn's remarks upon the greater ease with which the uncivilized get through the puerperal process remarked, that the difference in the anatomical formation of the pelvis conduces as much as anything else to this advantage.

Dr. Miller spoke of obliquity of uterus as a condition where manipulation was called for. Where the anterior lip is pushed down and pressed against the pubis, we have to interfere and correct this condition by artificial means. Is not in favor of giving ergot in the first stage of the labor on account of the continous pain produced being dangerous to the life of the child, and then, too, artificial dilatation will produce the action that is desired to be promoted by the ergot. Where by rupture we lose the valuable assistance of the membranes, and we have not that fortunate occurrence when the scalp forms itself into a sort of pouch and takes their place, artificial means may well be resorted to. Has used injections of warm water into the rectum as a stimulant to the womb. Does not agree with Dr. Taliaferro as to the length that the first stage may continue; is not in favor of forcible dilatation in any stage, but would interfere to prevent the first stage being too tedious.

Dr. W. F. Westmoreland has had but little experience in obstetric practice; has always felt, however, that it was the duty of the physician to save his patient of all the pain possible. If artificial dilatation hastens labor, and thus shortens this trying ordeal, he could see no objection to it.

Dr. Miller remarked that he usually dilated during a pain, as otherwise the contraction produced is very irregular. Does not think there is any danger of rupturing the membranes by manipulating with the finger.

Dr. Taliaferro thinks there is danger of rupture of the membranes in manipulating with the fingers during a pain. He does not wish to stimulate the uterus to increase contraction by artificial dilatation, or by other means in the *first stage* of labor. Would prefer if the pains are tedious to arrest or modify them with an opiate, and give the patient an interval of repose with the view not alone of obtaining a period of rest for the patient, but of increased activity in the contractions when in some hours they are renewed.

Dr. Todd, in response to a request from Dr. Miller as to his views, agreed with Dr. Taliaferro. A rigid os could not be dilated with a finger, lax os needed no interference. Tart. antimony, chloroform, and then the lancet, were his weapons to overcome rigidity. Thought the danger of rupture of bag of membranes counter-balanced any problematical hastening of delivery by such interference. Coincides heartily with Dr. Taliaferro's suggestion of morphine to quiet the patient when the first stage of labor is tedious.

J. S. TODD, M.D., REPORTER.

---

ATLANTA, GA., May 30, 1876.

The President, Dr. H. V. M. Miller, in the chair.

Dr. J. G. Westmoreland opened the discussion of the evening, by inquiring of the Academy their several opinions as to the treatment of epidemic or malignant dysentery. Remarked that there were a good many cases of dysentery in town now; that the disease was earlier this year in making its appearance than usual. Said he had been through several epidemics, and the same treatment that was given in sporadic cases would not answer when he had to encounter the malignant form of the affection.

Dr. J. P. Logan had never been so unfortunate as to encounter dysentery in its malignant form, but had, of course, seen violent sporadic cases. His treatment was: When the flux was preceded by diarrhœa, to rely entirely on opium, and has generally cured such cases in from twenty-four to forty-eight hours. When bowels have not been thoroughly moved, he evacuates them with a saline cathartic, and then resorts to opium. He believes most of the severe cases which he has seen to be the result of neglect in their incipiency. Theoretically, no remedy is apparently more indicated by the pathology of dysentery than mercury, and when he first began to treat the disease—in fact for a long while—he gave calomel, but has now entirely abandoned its use. He rather doubts the existence of maliglant dysentery as an epidemic.

Dr. Armstrong agreed with Dr. Logan in his treatment. The cases which he has encountered this year were all mild; has never seen malignant dysentery as described by the books. He particularly objects to his patients taking draughts of fluids of any kind while under treatment. Thought sweet milk the best diet, preferred its being taken warm and in small quantities at each potation.

Dr. J. G. Westmoreland learned in the epidemic through which he had passed, that if opium arrested the affection it was not malignant. He gives blue-mass and opium, and frequently uses astringents and opium in mucilage per anum (always after defecation,) with good result. In severe cases he considers a fly blister over the abdomen of the highest importance. He thinks small doses of mercury injurious. He gives a large dose at first, and then leaves it off entirely. During convalescence he has known frequent relapses from a single meal of fresh meat. In malignant dysentery it is very essential to nourish your patient.

Dr. Taliaferro thought the opium test of Dr. Westmoreland correct. Many cases recover spontaneously, others are cured by a simple purgative, but when symptoms are alarming, and the cases do not take this favorable turn, he relies on small doses of calomel and opium, repeated every four or six hours. He has been much pleased recently with thirty-grain doses of ipecac. given according to instructions of Dr. Woodhull, but when the ipecac. fails, he resorts to calomel in small doses with opium.

Dr. J. T. Johnson treats the disease by giving Rochelle salts, followed by opium. Does not like mercury. Is much pleased with and frequently practices medication per anum. Has used in chronic dysentery forty to fifty grains solution nitrate of silver to the ulcerated and inflamed rectum with satisfaction.

Dr. Rauschenberg was in an epidemic of bloody flux in 1851, that was very fatal. The cases that were treated with small doses of calomel and opium were very fatal. He began to treat with large doses of calomel, followed by castor oil until the bowels were thoroughly emptied of all irritating matter; then gave large doses of opium frequently repeated. If this did not speedily stop the discharges, added acetate of lead to the opium. He considered it of vital importance to bind up the bowels for twenty-four or thirty-six hours. When he succeeded in doing this, he considered his patient safe; would not hesitate to produce narcosis to effect this result. He had bled when the pulse was high, the skin hot, etc. Likes the nitrate of silver locally applied in the chronic form.

Dr. J. S. Todd thought ipecac. in mild cases a good remedy, and was pleased with its effects, though the first and only case he ever lost was treated with that drug and opium. In 1869, in private conversation with Dr. S. H. Dickson in his office, at Philadelphia, he showed him a book in which he had tabulated the results of treatment in eighty thousand cases. His information was gathered from every available source, and included every variety of treatment—large doses of ipecac among the rest. The conclusion from these statistics was that under calomel and opium, from first to last, the duration of the attack, including the time spent in convalescence from ptyalism, occasionally produced, was seventy per cent. shorter than under any other treatment, and there were seventy per cent. less fatal cases when these medicines were administered. Such an accumulation of statistics, from such a man, made an indelible impression on his mind. He had treated dysentery according to this plan: He places special stress on enjoining the patient to forbear from straining while at stool. Owing to the extensive derangement of the digestive function, considered hypodermic medication very essential. In protracted cases he thought quinine indispensable. Regards a bilious operation as the forerunner of convalescence, and had rather have his patient have one than bind up his bowels.

Dr. J. T. Johnson exhibited a specimen of calculus of the phosphatic variety, extracted by himself from a youth of sixteen or eighteen years of age, by the ordinary lateral operation, with bistoury. The patient recovered.

Academy adjourned.

---

ATLANTA, June 6th.

Dr. H. V. M. Miller in the chair.

Dr. Calhoun exhibited a specimen of an enucleated eyeball, and remarked that he reported the case simply to show what a length of time a foreign body could remain within the eye before any symptoms of a serious nature manifested themselves. The patient, now aged twenty-nine, living near Montgomery, Alabama, while out hunting nine years ago, had a piece of a gun cap to fly forcibly against the centre of the cornea, and penetrating this, lodged somewhere in the interior of the ball. The pain and subsequent severe inflammation subsided after a time under the ordinary anti-phlogistic treatment, but all vision, and even all perception of light, were lost to the eye. The ball was very much reduced in size, and now and then the patient would attempt to wear over it an artificial eye, but was forced to discontinue it on account of the irritation set up each time it was inserted. Eight and a half years after the accident, a small fistulous opening occurred in the cornea at the point at which the piece of cap entered, from which the watery contents of the eye gradually escaped, and the ball collapsed, being during this time, and subsequently, in a state of constant irritation. About four months ago, symptoms of sympathetic inflammation began in the formerly sound eye, and vision was gradually diminishing, with occasional attacks of severe pain. One week ago the diseased or injured ball was eneucleated in the usual way, from the effects of which he is even now almost entirely recovered. The sympathetic symptoms in the other eye have also disappeared, and the vision has almost assumed its normal condition. Upon cutting open the ball after its removal, in the centre of the anterior chamber a large piece of cap was found, having been in the eye a little more than nine years. That such a foreign body should have remained imbedded in the interior of an eye for so long a time without having given intense suffering is indeed strange.

Dr. Calhoun also reported a rare form of disease, viz: an abscess in the orbit (suppurative cellulitis) in a child five months old. When called to see the child, the left eye presented externally all the symptoms of beginning purulent ophthalmia, viz: the lids were enormously swollen, the upper overlapping the lower, and presenting the peculiar glistening appearance seen in purulent inflammations of the eye occurring in children. The little patient seemed to be suffering quite a a good deal, and had a high fever. A large leech was applied to the upper lid, and the bleeding kept up by warm cloths after it had fallen off, thus taking in all about an ounce of blood. Iced cloths were afterwards applied at intervals during the night, and on the following morning the swelling was found very much reduced, and the fever entirely gone. There was nothing more than a little irritation of the conjunctiva, which, with the remaining swelling, passed off in a few days under the use of warm cloths and the application of a weak astringent. At the end of four or five days the patient was dismissed, but two days afterwards I was again called, and found the swelling of the lid had again taken place during the previous night, but not so much as before; nor was there that redness present as in the first instance. The conjunctiva was still free from inflammation (only irritated), and the child had very little fever. The ball, however, was pushed downward and forward, causing a considerable displacement. The same treatment was applied, but with only partial success. The lid gradually diminished, but in same proportion a greater protrusion of the ball took place. I was satisfied that an abscess had formed in the orbit around the ball, and after a day or two more was enabled to detect a little fluctuation. A narrow bistoury was passed through the upper lid behind the ball, fully to the depth of three-quarters of an inch before the pus was reached, which flowed out very freely to the amount, perhaps, of a teaspoonful. A tent was placed in the wound, and the pus evacuated several times daily, with the effect of the entire disappearance of the swelling and the return of the ball to its natural position. Matter is still being discharged from the wound, but everything is favorable for the complete recovery of the child. The great danger in all such cases is the extension of the disease into the brain along the periosteum and through the optic foramen, forming frequently an abscess in the base of the brain. In the adult suffering with abscess of the orbit, the conjunctiva of the ball and lids is very much inflamed and swollen, frequently overlapping the cornea in a wall-like circle. In this case no swelling or inflammation of the conjunctiva took place at all, nor was there any fever after the first night, also an unusual occurrence in such diseases.

There was no apparent cause for the formation of the abscess, aside from the child having taken cold. During the treatment the other eye showed symptoms of taking on the same disease, but passed off in a day or two without amounting to anything.

Note.—Since reporting the above case at the last meeting of the Academy, until Friday the symptoms gradually diminished; the pain was less, ball had receded, no fever. Friday he inserted a tent; during the night some evidence of pain in the head, but not much on the following day. Saturday it went into convulsions, on the side opposite the affected eye; on that side paralyzed. The pupil was continuously widely dilated. Small doses of chloral controlled the spasms. The child died in a few hours. He is inclined to think, from present information and sequences, that the brain was primarily affected—so little, though, that it escaped observation until the occurrence of the spasms. He did not see the child after Friday, Drs. Miller and Taliaferro being in attendance at its death.

Dr. Baird reported a case of tertian intermittent fever cured by sulphate of cinchonidia, recently sent him by Powers and Weightman. Patient had suffered for four or five years with malaria, the system being saturated with it. The case was also complicated with dysentery. He gave it to this case in five grain doses until twenty grains were taken, last dose four hours before chill. Second day same dose; is now giving two grain doses ter die as a prophylactic. Its cheapness as compared with quinine, and the high testimonials which it bears, entitle it to consideration.

Dr. Todd had used it with success in one case of intermittent fever where quinine had failed to arrest two paroxysms. It was not a new alkoloid of cinchonia. He thought well of it, and the sulphate of cinchonia.

Dr. John M. Johnson wished further to define his views with regard to the therapeutic action of the muriate of ammonia on fibroid tumors. He believed the remedy most potent agent at our command to discuss all tumors having for their being fibrin and were the result of the deposition of inflammatory product. Enlargements of the spleen, goitre, and even the cysts of ovarian tumors, were modified and cured by this drug. As an instance, a woman, a negress, presented herself to him for treatment, having what he and Dr. Miller diagnosed to be multilocular ovarian tumor, occupying the entire cavity of abdomen. She was placed on sacch. colomel at night, and muriate of ammonia three times daily in fifteen grain doses. She also wore a bandage around the abdomen, kept constantly wet with the sal-amoniac. The treatment was begun two years ago. He saw her to-day at his office, she is as slim as a race-horse; the remains of the tumor are imperceptible to a casual observer.

Second Case.—A tumor on side of the womb, supposed to be fibroid. Under treatment four months. It is now about half the size as when treatment was begun, or about as large as a goose egg. In answer to inquiry from one of the members, he stated that when the tumors was on neck or mouth of womb, or in the nasal passage where they could be inspected, he also used the muriate tincture of iron locally applied, as stated in his article in the February number of the ATLANTA MEDICAL AND SURGICAL JOURNAL. His general dose was from fifteen to twenty grains ter. die. He would expect to cure a fibroid so situated, in from two weeks to six months. He also mentioned a fibroid on posterior hip and an elongated cervix cured by this procedure.

Dr. Taliaferro asked him if the fibroid in the abdomen, which had diminished so rapidly, was attached to the uterus.

Dr. Johnson replied that he did not know positively, it may have been attached to the broad ligament. He was certain of one thing, that it was a fibroid tumor. He mentioned a case of painless pneumonia which had also been treated by Dr. Sayre, of New York. There was complete hepatization of one lung, which condition had existed for some time. Under the muriate of ammonia, internally and externally, in two months time the patient entirely recovered, but died two years after with tuberculosis of brain.

Dr. J. T. Johnson knew a gentleman recently to recover where no air entered the lungs, without having taken any muriate of ammonia. Dr. J. M. Johnson knew the gentleman to whom he alluded, but diagnosed his affection capillary bronchitis, cured by expectoration.

Dr. Miller said the muriate of ammonia was a remedy highly esteemed, and long used by him, for the following reasons: It was admissable of direct chemical proof that it diminished the amount of fibrin in the blood one-half; it prevented, when added to freshly drawn blood, its coagulation, and when a clot is allowed to form the application of any preparation of ammonia to it would re-dissolve it. These experiments made by Dr. Richardson, of London, and also by Dr. Jones, of the Medical College of Georgia, established these facts. Accepting these experiments, the rationale of its action was apparent. The number of diseases in which the fibrin is increased in the blood is large, in fact all inflammatory disorders, and some non-inflammatory. Our principal agents to defibrinate the blood, to discuss tumors, to cut short inflammation, were mercury, antimony, iodine, and, with him prominently and preferable to the others, ammonia; better, because there was no danger of ptyalism, there was not that nausea and depression consequent upon the administration of tart. antimony, and the constitutional disturbance sometimes manifested by long courses of iodine — prominent among which were cutaneous eruptions and faucial irritations. In a long spell of sickness we have all witnessed the emaciation of persons so suffering. This loss is caused principally by the absorbents taking up the adipose tissues. In hydrops, we give saline or hydrogogue cathartics, diuretics, to render the blood less fluid, nature supplies the matter thus abstracted from the blood, by utilizing the effusions wherever they exist. Following up this course of reasoning, he gave ammonia to cure abnormal enlargements when such are dependent on fibrin deposits. He defibrinizes the blood, nature always tending to equilibrium, seeks to render that fluid normal, by taking up the fibrin *most recently* thrown out. He could multiply Dr. Johnson's citations almost indefinitely, illustrated by cases which he had treated, all bearing testimony to the practical truths of the theory given. He once had under observation, at the same time two cases of goitre. One he put on iodine, externally and internally; the other muriate ammonia, externally and internally. After one month, the one on iodine was better, tumor softer, but little diminished in size, the other almost entirely well. The iodine was discontinued in No. 1, and the cure perfected with ammonia. Knows of a case of goitre so treated ten years ago, where the cure has been so complete that you cannot detect there ever having been enlargement in the gland. In contusions, applied externally, he knows of nothing so good. Has cured many enlargements of the uterus with it. Has given it for six months continuously, thus showing the length of time it may be given without detriment to health. It would, when applied to a whitlow circumscribe the inflammation. Can't see how it would benefit in neuralgia, unless it was of rheumatic origin. He gave it in acute rheumatism, but would not say that it cut short the disease, as he rather thought rheumatism was a fever which had a certain course to run. However, he always gives it, on account of the large increase of fibrin, which is often as high as ten parts in the thousand, and has seen such good effects from its administration, that he occasionally thought it had much to do with abridging the duration of an attack. Cancerous, scrofulous, and syphilitic deposits are heterologous formations; the ammonia would have no effect on them, and when such tumors were lessened in size during its use, it was not owing to their peculiar exudations being taken up, but attributable to the absorption of fibrinous deposits, the result of inflammation, caused by their presence in the tissues. He never gave carbonate of ammonia in low or anæmic states of the system, because he did not wish to give a remedy which would defibrinate the blood, and thereby further impoverish the already weakened circulating medium. He had never known a case to recover, when carbonate of ammonia was given, the circumstances being as above stated. Prof. Jones, of the Medical University of Georgia, has shown that in typhus and typhoid also, that ammonia was present in the blood, and acted as a destroyer, by causing early decomposition of that fluid.

Dr. Connally had used the ammonia chlo., in conjunction with ergot, in one case of enlargement of the uterus. The organ was so large that it changed position in the pelvis with corresponding movements of body. The patient's age was forty. She had leucorrhœa also.

The ammonia was given one week and then for some period of time ergot, thus alternating. In six weeks the womb had reduced below the normal standard.

Dr. Taliaferro thought ergot did more good than the ammonia in that case. He asked Dr. Miller would ammonia influence hypertrophy of muscle.

Dr. Miller said hypertrophy is development and not deposit. Muscular fibre and fibrin are different. In atrophy of muscle from disease of disuse the fibre was not absorbed, no essential characteristic part of it was lost, in other words, shrunk, but retained its peculiarities; the fibres were the same in number before the enlargement as before.

Dr. Taliaferro differed with Dr. Miller in his pathology, contended that enlargement was not dependent on fibrinous deposit, and was not a result of inflammation, but caused by super-nutrition in the areola or cellular tissue was the pathology of the enlargement to be found. The enlargement or growth of the womb during pregnancy was muscular, largely due to new muscular fibres, which by fatty degeneration during the process of involution are entirely lost.

Dr. Miller said that if enlargement of womb was not dependant on fibrinous deposit then the remedy did more than he thought for. The uterus during pregnancy, according to his pathology, had no additional muscular fibres; those in it were only hypertrophied, that is, increased in size and strength.

Dr. J. M. Johnson thought Dr. Miller wrong when he gave no ammonia in asthenic fevers, especially typhoid. In that disease he gave it to modify and prevent the inflammation and sequences on Pyer's glands, to preserve the normal fluidity of the blood, and for its peculiar stimulant action.

Academy adjourned.

